# Hudson River Estuary sediments contain high fractional abundances of preserved globothalamean foraminiferal organic linings at Piermont, New York

**DOI:** 10.1371/journal.pone.0361863

**Published:** 2026-10-30

**Authors:** Logan Brenner, Laura Haynes, Batoul Saad, Maisie Klem, Amelia Lang, Elizabeth Grey Linsley, William Riley, Sophie Spink, Carolyn Zhao, Margie Turrin, Lorelei Curtin

**Affiliations:** 1 Department of Environmental Science, Barnard College, New York, NY, United States of America; 2 Department of Earth Science and Geography, Vassar College, Poughkeepsie, NY, United States of America; 3 School of Earth Sciences, The Ohio State University, Columbus, OH, United States of America; 4 Lamont-Doherty Earth Observatory, Palisades, NY, United States of America; 5 Department of Geology and Environmental Geosciences, Bucknell University, Lewisburg, PA, United States of America; Tsinghua University, CHINA

## Abstract

Foundational to an estuarine ecosystem is the composition of its sediments, with benthic foraminifera serving as a key component. While it is common to find intact and deformed tests, foraminiferal organic linings (FOLs; foraminifera whose tests dissolved) are not always present in sediment samples. Identifying where FOLs are preserved is a necessary first step towards determining what promotes their development and burial. Here we document the prevalence of globothalamean FOLs in the Hudson River Estuary (HRE) at the Pier in Piermont, New York, USA. We also identified six agglutinated foraminifera species and successfully imaged foraminifera that were collected while in the process of losing their tests. There are a multitude of factors that can lead to test dissolution with FOL preservation, but most are related to suboptimal growth conditions. While the HRE has a history of sediment pollution, studies utilizing foraminifera or FOLs to study the modern benthic environment are not common. Our study provides some of the first evidence for FOLs preserved in the HRE. This exploratory work is a critical step towards utilizing foraminifera as a proxy for benthic conditions, with specific recommendations for future FOL sampling to monitor sediment conditions in the HRE.

## Introduction

Estuaries are critically important ecosystems, often existing at the intersection of the natural and human-built environment. They are ideal locations for urban development, providing transportation channels, recreation sites, support for agriculture and aquaculture, and diverse ecosystems both in and adjacent to the water. In the United States, counties with estuarine regions comprise only 4% of the country’s landmass but produce 47% of the economic output and 37% of employment [1]. The Hudson River Estuary (HRE) is no exception, with an intimate relationship to the development and maintenance of New York City and humans relying on its ecosystem services [2,3]. Projected climate change, however, presents a serious threat to HRE ecosystems via warming, deoxygenation, and sea level rise [4–7]. In addition, the HRE is susceptible to pollution from sewage, industrial waste, fertilizer, and physical manipulation of the estuary waterway [8,9]. Therefore, understanding the biogeochemical status of the HRE is critical to ensure its future, is a necessary act of stewardship, and will allow humans to continue to reap the important services the estuary provides.

*In situ* measurements of water and sediment, surveys of marshlands, as well as qualitative and quantitative measurements of macro-organisms, such as fish, have proven to be incredibly valuable [10–12] for understanding the status of the HRE. To fully evaluate the HRE, or any estuary, its sediment composition must be thoroughly understood. In particular, a census of the benthic foraminifera population can provide unique insight into the estuary [13–29] and can constrain the biogeochemical and physical parameters of the foraminifera’s environments, including temperature, salinity, and water quality [30–37].

In the whole of the HRE, previous investigations divided foraminiferal assemblages into three broad categories, essentially by salinity. Note that salinity values presented here are on the Practical Salinity Scale (PSS-78) and are thus dimensionless. Assemblages dominated by calcareous foraminifera are present in the southern portion of the HRE, from the 15 isohaline, at approximately the intersection of the Harlem River (~21 km north of the New York Harbor) southward to the New York Bight and Atlantic Ocean [38–40]. Agglutinated foraminifera dominate assemblages from the 15 isohaline northward to the 5 isohaline, to approximately Piermont, NY [38–40]. The third assemblage type is considered more cosmopolitan, containing substantial portions of both agglutinated and calcareous foraminifera, located from the 25 isohaline northward to the 12 isohaline [38–40]. These previous studies illustrated how assemblages vary on a macro scale using sediment cores tens of kilometers apart and are largely controlled by the salinity gradient along the HRE.

However, these assemblage studies did not assess the prevalence of foraminiferal organic linings (FOLs) in HRE sediments. FOLs, typically brown to golden in color, are acid resistant (i.e., they can be preserved in low pH conditions), composed of proteins and polysaccharides, and represent foraminifera whose tests have dissolved. FOLs can remain preserved in sediment even when an agglutinated test, which can be held together by an organic and/or calcareous cement, or calcareous test dissolves [41–43]. Dissolution of the test can be caused by dysoxia/hypoxia or low pH, which can be due to increased organic matter respiration in sediments or pollution leading to increased acidity [15,43,44]. In addition, cable bacteria, which are sulfide oxidizers, have been implicated in estuarine sediment acidification and associated dissolution of foraminifera tests [45–47]. Thus, FOLs may exist in response to many important characteristics of a benthic ecosystem and their presence should be considered in any modern benchmark foraminifera census.

Here we present an exploratory study to provide baseline observations on the presence of FOLs in the HRE. We studied six core top sediment samples from Piermont Pier in Piermont, New York, USA to better understand the modern HRE foraminifera assemblage and specifically document the presence of FOLs. At ~40 km north of New York City and the New York Harbor, estuarine conditions persist at Piermont. As a prominent feature in the town of Piermont, the Pier has a rich history, including serving as a railroad station and the former site of a paper mill. Today, the Pier is largely used for recreational activities as well as academic research, outreach, and community science initiatives owed to the presence of the Lamont-Doherty Earth Observatory Field Station. Therefore, in addition to the natural development of the HRE system, the historical and modern activities on and surrounding the Pier may impact the benthic ecosystem and the presence of FOLs. We found that FOLs dominate the foraminifera assemblage, otherwise comprised of agglutinated foraminifera.

## Materials and methods

### Study site: Pier at Piermont, New York, USA

The Pier at Piermont is situated on the western bank of the HRE, located ~40 km North of the estuary’s Atlantic Ocean outflow point. The Pier experiences semidiurnal tides with a tidal range of ~1 m. It should also be noted that Piermont Pier functions primarily as a jetty being built directly into the HRE bottom. Only the terminus of the Pier has a traditional pier appearance, supported by pilings (Fig 1). This is important to acknowledge because jetties have been shown to reduce the velocity of currents, decrease seawater intrusion to the side further from the ocean, and reduce lateral sediment stratification in estuaries [48]. The bottom habitat surrounding the Pier is dominated by soft silt and clay. The vegetation surrounding the pier is dominated by *Phragmites australis* (common reed), an invasive species [12]. The Pier represents the northern boundary of the Piermont Marsh, which is an ecologically significant home to diverse wildlife, an important carbon sink, and is one of four tidal marshes in the Hudson River National Estuarine Research Reserve [11]. Mapping of Piermont Pier (Fig 1) was conducted using R (v4.6.1; ggplot, ggspatial, maptiles).

**Fig 1:**
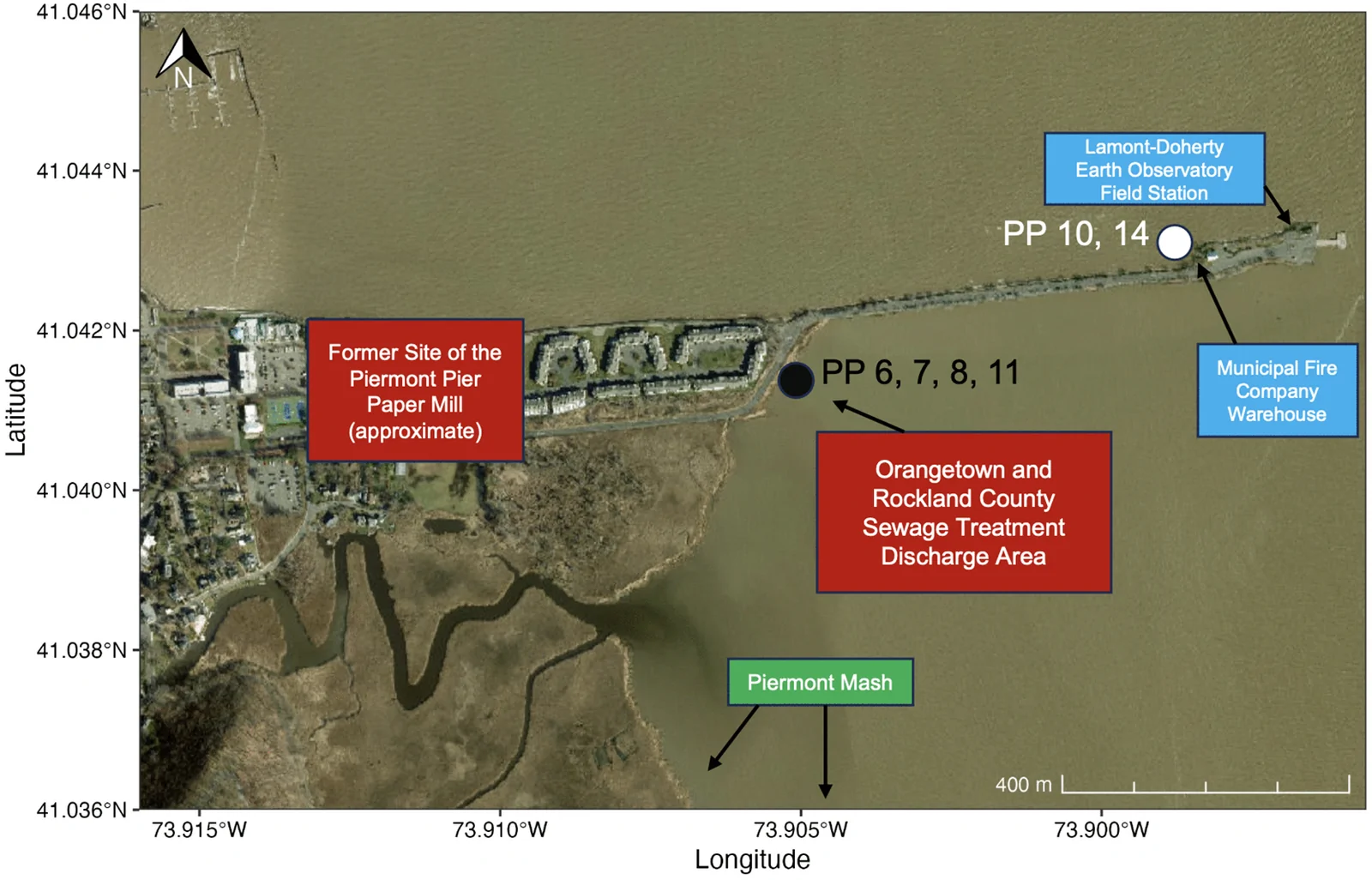
Map of study site: Piermont Pier in Piermont, New York. The black (PP 6, 7, 8, and 11) and white (PP 10 and 14) signify the core top locations. Satellite basemap tiles provided by Esri World Imagery tile service (provider = “Esri.WorldImagery” via maptiles R package), licensed under CC BY 4.0.

The Pier was originally built in the late 1830s CE and is about ~1.2 km long. The Pier once marked the end of the Erie Railroad and was a transfer point for train passengers to board boats bound for New York City. In addition, The Piermont Paper Company paper mill, constructed at the base of The Pier, began production in 1902 and benefitted from the pre-existing railroad. The Pier’s sociological significance continued, as it served as the transfer point for U.S. military troops leaving for the European Theater during World War II. The Piermont Paper Company continued production through WWII into the late 20^th^ century but officially ceased functioning in 1984. The paper mill was demolished and replaced by luxury buildings, homes, and restaurants. Pollution associated with Paper Mill activities likely included, Zn, Cu, Pb, and polychlorinated biphenyl (PCBs), amongst other contaminants, and the area was designated a State Superfund Site. As recently as 2017, 280 cubic yards of PCB-contaminated soil was removed through the New York State Superfund Program. While today the Pier is a recreation site with a storage building for the local fire company, the former paper mill, in conjunction with the railroad, and military industries, still serve as likely sources of regional legacy pollution.

Active outflow pipes from the Orangetown Sewage Treatment Plant and the Rockland County Sewage Treatment Plant, which are situated in the Sparkill Creek Watershed, are located on the south side of the Pier. These sewage treatment plants discharge ~24.5 million gallons of treated water per day nearby the Pier, representing the second largest discharge point of treated sewage in the HRE north of New York City [49]. The treated sewage water is still often high in nitrogen and phosphorous. In addition, raw sewage is also occasionally discharged into the Sparkill Creek Watershed, and subsequently into the HRE at Piermont Pier. Historically, this occurred because the treatment plants in Rockland County (NY) Sewer District #1 and Orangetown (NY) that operate in the Sparkill Creek Watershed can be overwhelmed when storm runoff enters the system [50]. During high rain events, old, porous piping allows water to infiltrate the system forcing the discharge of raw sewage and are therefore sources of heavy metals and an oversupply of nutrients that can result in a decrease of aquatic biological diversity [51,52].

### Instrumental data

All fieldwork was conducted in June 2023. *In situ* measurements of the physical and chemical properties of the water were not available, however instrumental data from the New York State Department of Environment and Conservation (DEC) were semi-continuously collected at the end of the Pier via grab sample (2013–2023) and are considered provisional. We note that these data likely do not reflect porewater conditions at each core site and are instead presented to provide insight into local summer conditions rather than attribute an environmental driver to FOL presence.

May-July averages for instrumental water temperature, DO, and pH values were calculated for the available sample period from the aforementioned New York State DEC. Due to equipment and logistical issues, measurements were not taken during the year of core sample collection (2023). Thus, available data for May-July water temperature, salinity, DO, and pH were only available from 2013–2016 prior to field work. Average water temperature and salinity during May-July was 21.8 ± 4.1°C and 4.5 ± 2.6, respectively. Average DO and pH were 8.4 ± 1.5 mg/L and 8.0 ± 0.3, respectively. While salinity was not measured by the DEC during our 2023 fieldwork, chloride concentrations were measured via Ion Chromatography. Both chloride and salinity data were available for samples collected from 2013–2016 and show a strong correlation (R^2^ = 0.99, p < 0.05, n = 58, S1 and S2 Fig). We utilized an ordinary least squares regression to quantify the relationship between chloride and salinity to calculate an estimate for salinity with the DEC grab samples from 2016–2017 and 2022–2023. Including the calculated salinities from our extended record, shifts the average May-July value to 6.0 ± 3.3.

### Sample collection

We retrieved a total of six sediment push cores by hand. Four cores were taken on the south side of the pier with the remaining two along the north side (Fig 1). All south side samples were taken in close proximity to Orangetown Sewage Treatment Plant and Rockland County Sewage Treatment Plant outflow. Due to the small number of cores and geographic range, these samples represent general Pier conditions, rather than provide specific insight into spatial variability. Cores were collected by wading approximately 1–5 m into the HRE at low tide; therefore, safe accessibility was a limiting factor when taking cores, hence the clustering of the locations (Fig 1). Samples were extruded and the top 1 cm was placed into 120 ml Nalgene storage bottles in the field. The core tops were divided, with half allocated for foraminiferal assemblage evaluation. The core top material that was reserved for FOL and foraminiferal evaluation was stained in the field with Rose Bengal (2 g per 1 liter 70% ethanol). These samples were kept in a cooler while in the field and at room temperature in the lab for two weeks prior to sample sieving and washing. Permits were not required for sample collection because of our affiliation with the Lamont-Doherty Earth Observatory Field Station.

### Foraminiferal evaluation: Sample washing and picking

Samples reserved for FOL and foraminiferal evaluation were washed and sieved prior to species identification. Each sample was washed with deionized water using a low-power pressurized pump sprayer through a stack of 3 sieves (2 mm, 250 μm, and 63 μm). The samples were washed for 10 minutes through the 2 mm sieve, 10–15 minutes through the 250 μm sieve, and another 20–25 minutes through the 63 μm sieve. The sediment air dried in the sieves for at least 24 hours at room temperature and was then stored in glass vials. To assess FOL abundance relative to intact species, we counted 100 individuals total (intact + FOLs) from the 63–250 μm fraction, a previously established sample size for identifying dominant populations [53,54]. For some core tops, we exceeded 100 individuals in order to complete evaluation of the entire picking plate (S1 Table). FOLs were not explicitly speciated to the challenges associated with interpreting their morphology (see section 4.1) [43]. Intact foraminifera were speciated and included *Ammoastuta salsa, Miliammina fusca, Ammobaculites sp., Trochammina inflata, Jadammina macrescens*, and *Haplophragmoides wilberti* (Fig 2). The focus of this study was to determine which foraminifera were present at Piermont (i.e., speciated individuals) and to provide documentation and description of the FOLs. We calculated fractional abundances with a focus on a dominant population, FOLs in this study. We provided preliminary insight into the foraminifera population, omitting fragments or ambiguous individuals, however, foraminifera whose FOLs were partially visible and thus were in the process of dissolution were put into the FOL category. Abundances were presented as fractional abundances, reported as percentages.

**Fig 2:**
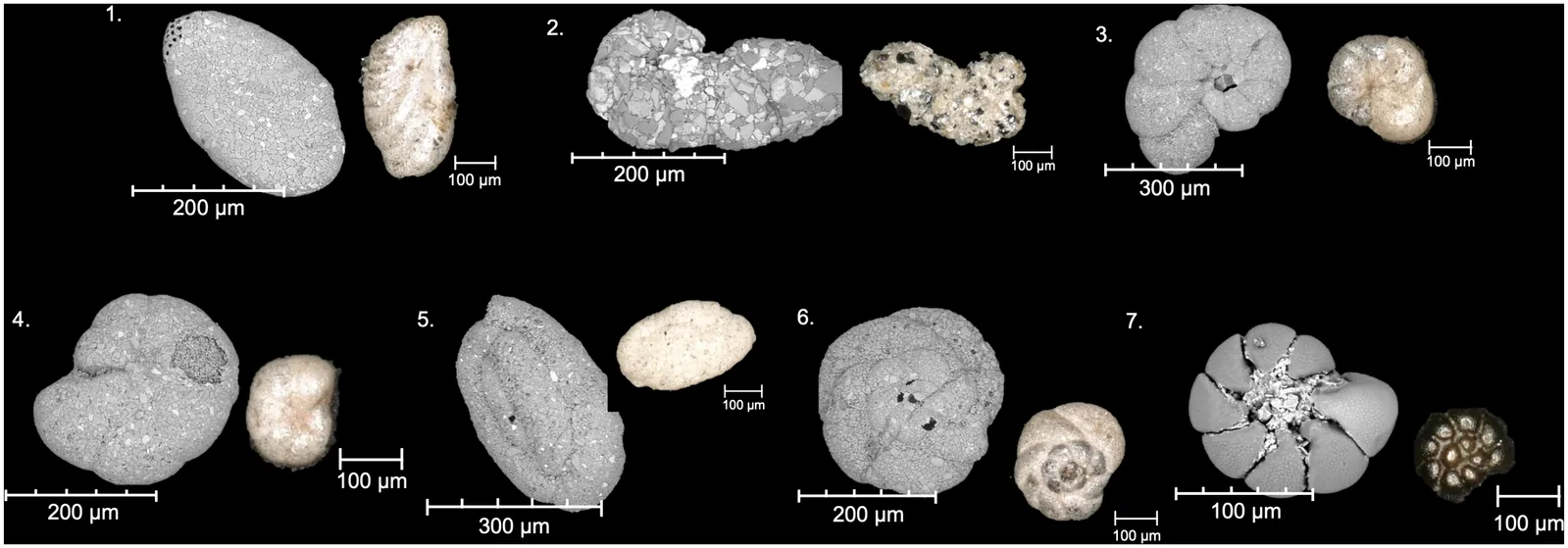
Microscopy images. Intact foraminifera: (1) *Ammoastuta salsa, (2) Ammobaculites sp, (3) Jadammina macrescens, (4) Haplophragmoides wilberti, (5) Miliammina fusca, (6) Trochammina inflata,* and (7) FOLs were imaged. Each panel includes SEM (left) and 3d (right) microscopy.

Our foraminifera counts were based on a combination of stained and unstained individuals. This approach was utilized to account for the prevalence of unstained foraminifera and modeled off of previous work. Eichler et al. (2007) [16] outlined modern foraminifera facies in an estuary in Brazil, which included *A. salsa, Trochammina sp.,* and *H. wilberti*, and their samples did not generate many stained individuals. The unstained foraminifera were considered dead, therefore, their assemblage evaluation was based on total counts (i.e., dead plus living) [16]. This is the protocol that we adopted since the focus of our study was documenting FOLs rather than developing a definitive assemblage.

We plotted assemblage data in stacked columns and generated an average assemblage with standard error bars. However, ecological data, such as foraminiferal counts, can exhibit high sample-to-sample variance, which can lead to inaccurately narrow confidence intervals when comparing FOLs prevalence. To account for this sample heterogeneity, we evaluated overdispersion in our samples, essentially to determine whether FOL variability exceeded what would be produced with a standard binomial model (i.e., FOLs vs. non-FOLs) and to generate a more statistically robust understanding of FOL presence. We first collapsed our data into two categories, FOL and non-FOL, using raw counts. We then fit an intercept-only Beta-Binomial Generalized Linear Model (GLM) with a logit link (i.e., log odds) using the glmmTMB package (v1.1.8) [55] in R (v4.6.0). We compared Beta-Binomial model results with a standard Binomial GLM to determine whether overdispersion was statistically present. We then calculated the overall population mean FOL abundance and its corresponding 95% confidence intervals using the inverse-logit function (plogis()). Lastly, we compared FOL abundances with non-parametric binomial 95% confidence intervals (Clopper-Pearson approach) to present the core-specific perspective.

Each foraminifera species and the FOLs were imaged with a 3D digital microscope (Keyence VHX-5000 Digital Microscope) at the Lamont-Doherty Earth Observatory. Further imaging was conducted using a Tabletop Scanning Electron Microscope (SEM, Thermo Scientific Phenom Desktop SEM) at Vassar College to further elucidate test morphology and structure.

## Results

Six core top samples in total were retrieved from the Piermont Pier study site (PP 6, 7, 8, 10, 11, 14) (Fig 1). Cores PP10 and PP14 were collected from the northern, upstream side of the Pier, in close proximity to the Fire Company storage building (Fig 1). The remaining four cores, PP 6, 7, 8 and 11 were retrieved from the southern, seaward side of the Pier, at the Sparkill Creek outflow point and in close proximity to a sewage pipe (Fig 1). FOLs, all with globothalamean morphology, were present in all samples (Figs 2, 3). In addition to the FOLs, we identified six agglutinated foraminifera species in each core top, *Ammoastuta salsa, Ammobaculites sp., Haplophragmoides wilberti, Miliammina fusca, Trochammina inflata,* and *Jadammina macrescens*, with no calcareous species present (Figs 2, 3)*.* All species were present in all core tops with the exception of *Ammobaculites sp.* being absent in PP10 and *M. fusca* absent in PP11. In all cores, either FOLs (PP 6, 8, 10) or *Ammobaculites sp.* (7, 11, 14) were most abundant in the assemblages (Fig 3). On average, the least abundant foraminifera was *M. fusca,* at 5.34% (Fig 3). We identified 27 globothalamean individuals that partially lost their tests, with a small portion of their linings visible (Fig 4).

**Fig 3:**
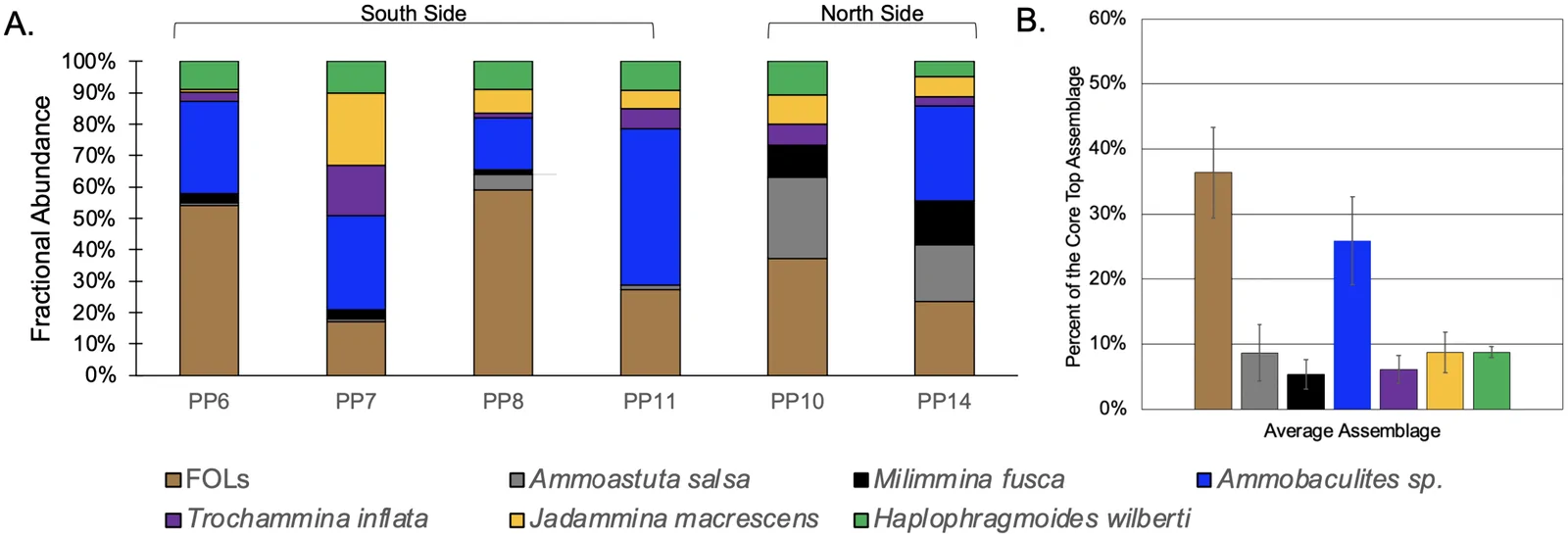
Core top results assemblage results. A) Foraminifera assemblage fractional abundance data, including FOLs, divided by core top B) Average core top assemblage (n = 6) with standard error.

**Fig 4:**
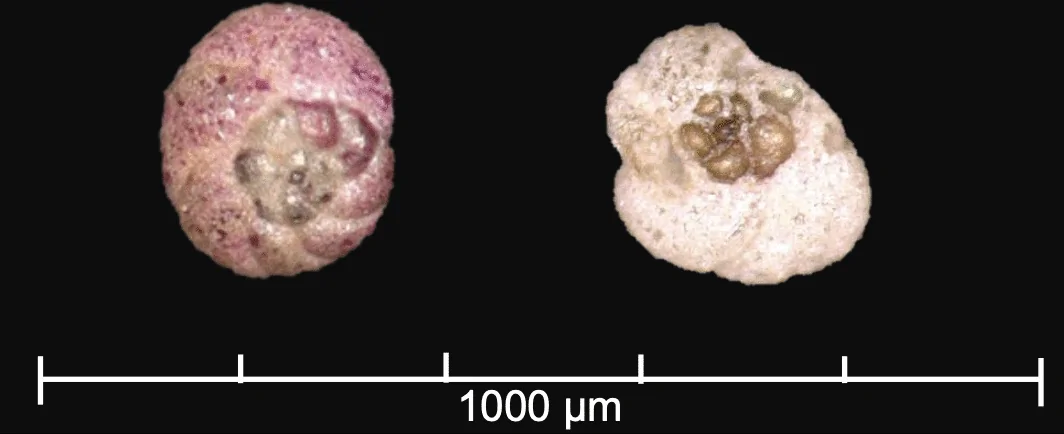
Foraminifera imaging. 3D microscopy images of *T. inflata* in the process of losing their tests to dissolution, bearing their FOLs that lined the more juvenile portions of their tests. Note the individual on the left is stained.

Of the 600 individuals counted, 188 were FOLs. The Beta-Binomial GLM baseline yielded a value of β_0_ = −0.78 ± 0.21 standard error (z = −3.64, p < 0.001) on the logit scale, back-transformed to a mean abundance of 31.4% (95% confidence intervals: 23.2%−41.1%). This value was only slightly smaller than the raw average of 36.4 ± 17.1(1σ)%. There was significant dispersion embedded within the dataset, with a dispersion parameter (ɸ) of 19 (Likelihood Ratio Test: χ = 20.91, df = 1, p < 0.001) that exceeded the expected output from the standard binomial model. We identified site-specific variations in FOL content, with a maximum value of 54% (95% confidence interval: 43.7%−64%) in PP6 that did not overlap with the mean value and a minimum of 17% (95% confidence interval: 10.2%−25.8%) in PP7 (Fig 5).

**Fig 5:**
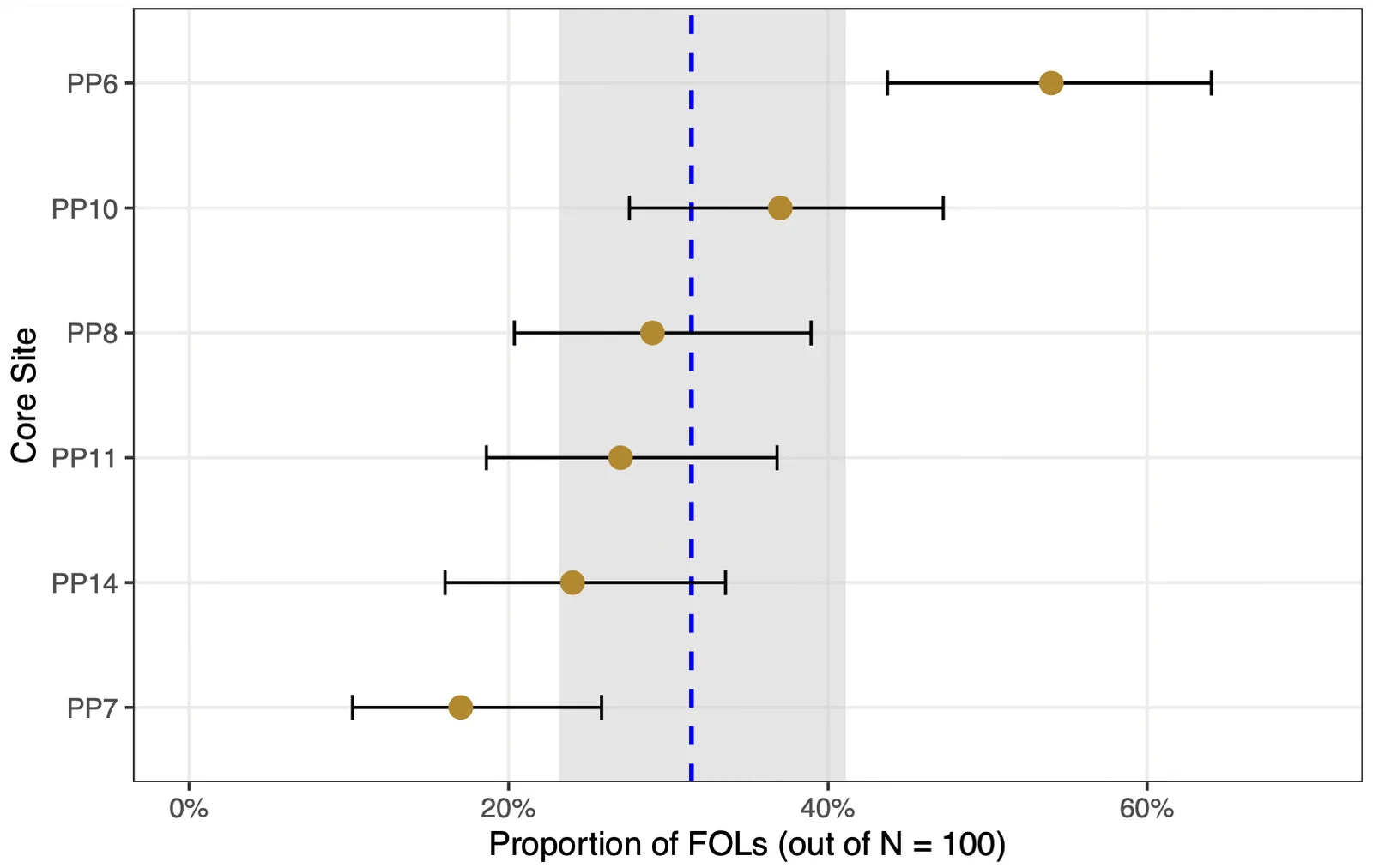
Site variability in foraminifera organic lining (FOL) abundance. FOL abundance (gold dots) by core top with exact 95% confidence intervals (error bars) calculated via the Clopper-Pearson method compared to the mean value of 31.4% (blue dashed line) with a 95% confidence interval of 23.2%−41.1% (grey shading), calculated with the Beta-Binomial model.

## Discussion

### The foraminifera assemblage in Piermont, New York

Each of our core top samples consistently yielded a diverse assemblage of between 5–6 agglutinated foraminifera species in addition to FOLs (Fig 3). However, nearly all foraminifera failed to retain the Rose Bengal stain, excepting a few *Ammobaculites* and *T. inflata*. We believe there could be multiple reasons for this outcome. Firstly, many of the agglutinated foraminifera in our assemblages were opaque, therefore making stained cytoplasm difficult to visualize. Related to this aspect of their morphology, it is also possible that they required longer immersion in the Rose Bengal stain. While some studies had success staining agglutinated foraminifera for short amounts of time, even 24 [56] to 48 hours [57], others preserved foraminifera in the Rose Bengal stain for 3–4 weeks [58] and even up to months [59]. Many of the foraminifera in our study (*A. salsa, M. fusca, H. wilberti,* and *T. inflata*) have successfully been stained in prior work [60], however this is not always the case. Similar lack of staining has been noted in other studies with agglutinated foraminifera. To that end, our unstained foraminifera may not have stained properly or were not living when collected. This was not delineated in our total counts, with the impact being that it is possible the assemblage did not reflect the truly local and current conditions.

Of the foraminifera identified, previous research indicated that *H. wilberti*, *A. salsa*, and *T. inflata* can be found at sites with similar salinity (mesohaline, 5–18 to oligohaline, 0.5–5), high bottom water temperature, a silt and clay (>80%) substrate, high organic carbon (9.1–9.6 mg/g), and low sulfur (2.0–8.2 mg/g) [16]. *M. fusca* have also been found in mesohaline and dysoxic environments [61] but can also thrive in areas with higher freshwater input, along with *T. inflata* and *H. wilberti* [62]. In general, all foraminifera identified in our study occupy niches with overlapping salinities. Due to the close proximity of the core tops and limited *in situ* data we do not make any assertions regarding environment type based on assemblage composition.

The intercept-only Beta-Binomial model produced an aggregate mean FOL value of 31.4% (95% confidence intervals: 23.2%−41.1%). This measure took into consideration the overdispersion inherent in the site-to-site heterogeneity [55, 63]. While prevalent, FOL abundance was still statistically below 50%. The maximum and minimum FOL fractional abundances were measured from nearby cores (PP6&7). Due to their close proximity, we do not attribute this variation to macro-scale environmental differences. Any spatial investigation of FOL abundance at the Pier will therefore require a much larger sample size with counts closer to n = 300 [54,64].

More generally, our results show that overdispersion does impact interpretation of foraminifera abundances in the HRE (ɸ = 19, p < 0.001). Cores in close proximity to one other may therefore not represent perfect random replicates and any reconstructions based on single cores are likely to be highly influenced by taphonomic variability. Thus, any future development of FOLs into bioindicators of the benthic environment should average many samples over a set geographic range and/or utilize hierarchical modeling, such as Beta-Binomial models [63].

### Implications of FOL presence

FOLs were abundant in every recovered core top with a baseline prevalence of 31.4% (Beta-Binomial-derived) (Fig 5). Prior studies of FOLs have indicated that their presence is often related to suboptimal benthic conditions that lead to test (calcareous or agglutinated) dissolution [43, and references therein]. In estuarine and other coastal settings, an increase in the acidity of bottom or pore waters, increase in the organic matter content of the sediments, or an increase in nutrient inputs are therefore often responsible for FOL presence [15,43,44]. The Pier is located just north of the Piermont Marsh (Fig 1) and export of the organic material that has built up over its ~ 5,700-year lifetime [65] could be impacting the composition of the Pier’s surrounding sediments. Increased organic matter loading from a combination of land use changes, landfills, sewage treatment plans, and runoff, amongst other sources are also possible at the Pier (see section 2.1). FOLs from agglutinated foraminifera have also been located in extreme marine environments where carbonate grains and tests are not preserved, notably the Challenger Deep in the west Pacific, which is at ~11 km and below the Carbonate Compensation Depth [41]. Finally, cable bacteria are sometimes associated with FOL presence, due to their ability to manipulate the pH of estuarine sediments [46,47].

We speculate that the prevalence of FOLs qualitatively indicates that benthic conditions at the Pier are conducive to foraminifera test dissolution. FOLs are worthy of further evaluation to fully uncover the impact of cable bacteria, bacterial decomposition, pH, pollution, or natural export of organic marsh material in the HRE. However, in this preliminary study the limited spatial coverage and small core top sample size, in conjunction with a lack of site-specific instrumental data, precludes definitive identification of FOL drivers and patterns.

### FOL speciation and its challenges

Speciation of FOLs is difficult as the linings are significantly smaller than intact foraminifera, an issue also noted by others [43]. The small size of the FOLs has been attributed to preferential preservation of the more juvenile components of the lining, owed to their thicker walls from the creation of multiple organic layers as compared to the adult chambers [43,66–68]. However, we were able to visualize a subset of *T. inflata* specimens that seemed to be in the process of losing their tests, baring their underlying FOL (Fig 4). FOLs with no preserved test are harder to speciate. FOLs identified as Trochamminacean species were also identified by Gooday et al. (2010) [41] at the Challenger Deep Site (>11 km depth). The FOLs imaged in the Gooday et al. (2010) study [41] were similar in appearance to what we recovered from our HRE site. We also identified pyrite framboids on some of our FOLs, which have been noted in damaged or dissolved *T. inflata* tests in previous studies [69] (Fig 6). The presence of framboids is of particular significance since pyrite develops in a reducing environment, often in organic-rich sediments with sulfate in pore waters. Therefore, the framboids serve as further, although not unequivocal, evidence of the low oxygen conditions that may lead to the degradation of foraminifera tests. All taken together, we speculate that *T. inflata* could be at least one, though not the sole, source of the FOLs preserved in our HRE sediments.

**Fig 6:**
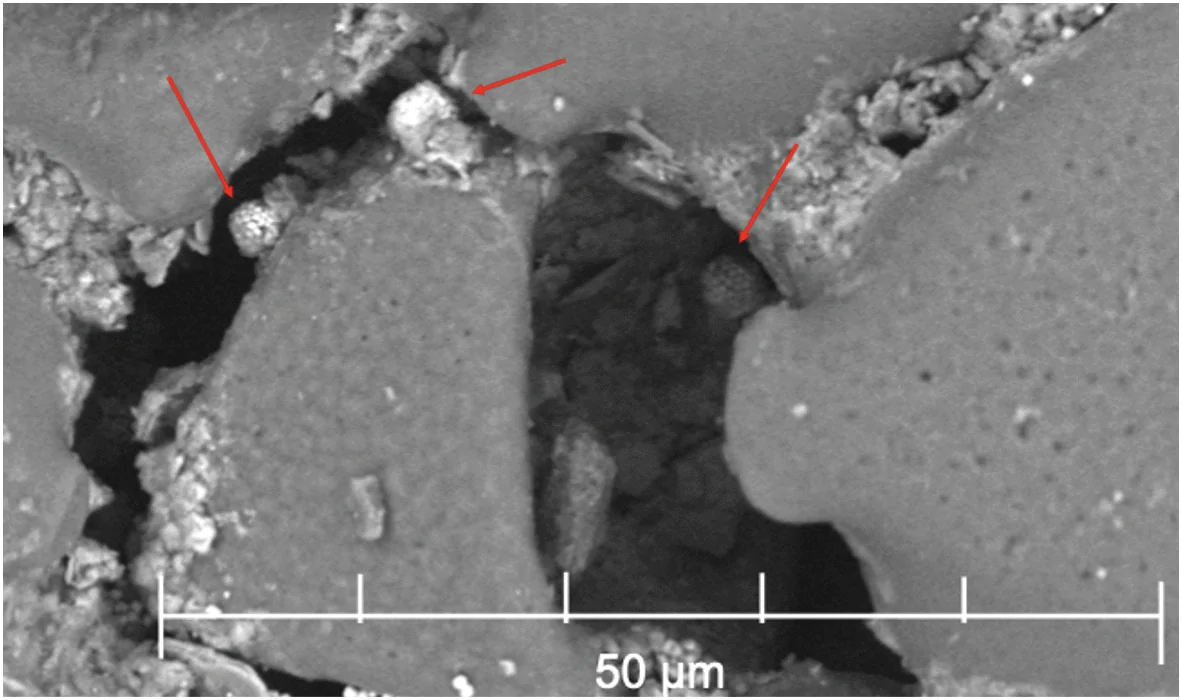
SEM image of FOL. Pyrite framboids within the FOL are noted by the red arrows. The presence of pyrite may represent low oxygen, reducing conditions.

An additional possibility is that FOLs represent the remains of dissolved calcareous foraminifera. While the complete FOLs have a trochospiral shape, the square chambers of the FOLs are not typically part of the *T. inflata* morphology and the presence of large pores are more likely to be indicative of calcareous specimens. However, no foraminifera with intact calcareous tests were present in any of our core tops. The June 2023 calculated salinity was 9.4 (S1, S2 Fig), a value that is typical of an agglutinated foraminifera assemblage as salinities less than 15 are thought to be intolerant for calcareous genera [40]. Nonetheless, the assemblage census study by Weiss et al. (1977) [39] identified *Ammonia spp*. as far north as Piermont. We speculate that it is possible that *Ammonia spp.* were present at Piermont seasonally and may have contributed to the FOL content of the foraminifera assemblages. Additional seasonal sampling and comparisons with *in situ* salinity measurements, as well as an updated estuary-scale foraminifera census, could clarify whether calcareous species were present at this location or indicate that assemblages shifted since the 1977 census.

### Recommendations for refining foraminifera investigations and the FOL proxy in the HRE

Our foraminiferal census at Piermont serves as a modern benchmark against which future studies can be compared. Cores that yield a deeper temporal history, beyond the modern snapshots of core tops, could also provide a comparison point prior to anthropogenic perturbations and deeper understanding of assemblage variability [40]. Collecting *in situ* data from specific core top sites, such as salinity, temperature, DO, and common measures of pollution (e.g., heavy metals, PCBs) of water and sediment will provide the insight needed to ascribe meaning to both temporal and spatial assemblage variability. For example, some of the species identified in our study, *J. macrescens, H. wilberti*, and *M. fusca*, have been considered tolerant to pollution [16,70] and instrumental data would allow us to tune the abundances of these species as proxies for contamination. Measurements of total organic carbon, total nitrogen, microbial indicators, or porewater pH will specifically aid in the interpretation of the conditions associated with FOL preservation.

The study of FOLs can be particularly helpful if one is evaluating the fossil record or when direct environmental measurements are not possible. However, further refinement of the use of FOLs as a proxy for benthic conditions in the HRE is also recommended before its full potential can be reached. In addition to the instrumental measurements recommended above, testing for the presence of cable bacteria [45–47] will help to isolate the drivers behind FOL presence and preservation. SEM with Energy Dispersive X-ray Spectroscopy could be used to evaluate the chemical composition of residues on the FOLs in an effort to determine whether they represent agglutinated or calcareous species. Speciation of FOLs could also be accomplished through Next Generation Sequencing approaches [71]. Finally, while the FOLs identified in this study were preserved well enough to be counted, dry picking can result in the destruction of more fragile individuals. Thus, we recommend the wet picking methodology for future studies focused on FOLs to ensure their preservation. Lastly, incorporating malformed or partially dissolved tests into assemblage counts could help inform interpretation of FOLs.

We consider these promising next steps for more comprehensively understanding HRE sediments. The species richness apparent in our samples surrounding the Pier warrant future in depth investigations as foraminifera assemblages and FOLs in the HRE hold great potential to serve as bio-monitors of the benthic ecosystem.

## Conclusion

The modern foraminifera assemblage from six cores taken proximal to Piermont Pier in Piermont, NY is composed of agglutinated foraminifera and FOLs. There are few published studies that evaluate distribution of foraminifera in the HRE, none of which focus on the presence of FOLs. Therefore, the work presented here represents an important preliminary investigation of the presence of FOLs. Considering their abundance, FOLs should be considered in biomonitoring studies as a tool for understanding the benthic ecosystem in the HRE. The drivers responsible for the preservation of FOLs are, however, still unknown and the mechanisms presented here serve as hypotheses to be explored. Future work should include spatially-precise ambient water measurements of environmental parameters, such as temperature, salinity, DO, pH, and pollutants. Partnering these instrumental data with more in-depth SEM microscopy and DNA sequencing will allow us to quantify the drivers behind FOL and intact foraminifera abundance and tune their assemblages and abundances as proxies for ecosystem conditions.

### Limitations

The work presented here should be considered an initial investigation, with the main purpose to officially document the presence of FOLs at Piermont, NY in the HRE. Due to the exploratory nature of this work, there are some limitations that preclude certain assertions. Namely the small sample size and limited geographic representation around the Pier limits our ability to provide insight into spatial variations in foraminifera assemblage. Further, the total foraminifera counts, based on stained and unstained individuals, limits our confidence in discerning the living, local benthic community from individuals who may have been transported and buried. Also, without *in situ* measurements from core sites we can only provide insight into general pier conditions with publicly available instrumental data. Due to this lack of site-specificity, we refrain from proposing an unequivocal driver of FOLs in the HRE at our study site and instead provide a few hypotheses to be pursued in the future. Conducting this study allowed us to develop a series of recommendations for studies focused on FOLs and interpreting their presence and preservation*.*

## Supporting information

S1 Fig Relationship between measured Chloride and Salinity in DEC grab samples at Piermont Pier from 2013–2016 with an ordinary least squares regression (n = 58) describing the relationship (p < 0.05). (TIF)

S2 Fig Salinity and Chloride Concentration at the Piermont Pier from 2013–2023 from the DEC water monitoring program. From 2013–2017, Chloride and Salinity were both measured. From 2021–2023, measured Chloride was used to calculate Salinity using the relationship in Figure S1.
(TIF)

S1 Table Raw counts of foraminifera and FOLs divided by core. (XLSX)
